# Dr. James Irving Rasbach

**Published:** 1912-05

**Authors:** 


Dr. James Irving Rasbach, of Ilion, died March 27, 1912, of cerebral haemorrhage, aged 59. He was a graduate of Bellevue Hospital Medical College, 1876. For 18 years, he had been a member of the local board of education.

